# Nurses' Handover Satisfaction: Development and Validation of the Handover Quality Questionnaire

**DOI:** 10.1111/wvn.70098

**Published:** 2025-12-28

**Authors:** Mayra Veronese, Elisabetta Cesaro, Lucia Stivanello, Chiara Daicampi

**Affiliations:** ^1^ Department of Cardiac, Thoracic, Vascular Sciences and Public Health University of Padua Padua Italy; ^2^ Department of Medicine University of Padua Padua Italy; ^3^ Healthcare Profession Department Padua University Hospital Padova Italy

**Keywords:** continuity of care, handover quality, nursing handover, patient safety, psychometric validation

## Abstract

**Background:**

The handover process is a critical component of patient safety, enabling effective communication and the transfer of responsibility among nurses. However, despite their critical role, it is often compromised by interruptions, lack of standardization, and variability in practice, which may reduce quality and nurse satisfaction. Existing tools primarily measure information transfer and efficiency but rarely incorporate nurses' perspectives on safety and satisfaction. This gap underscores the need for a validated instrument that comprehensively assesses handover quality from both a professional and safety perspective.

**Objectives:**

To develop and validate the Handover Quality Questionnaire (HAND‐Q), a tool assessing nurses' satisfaction with handovers and their perceived impact on patient safety.

**Methods:**

HAND‐Q development included a conceptualization phase (literature review, expert discussions) and a validation phase (pilot and large‐scale testing). Exploratory and Confirmatory Factor Analyses (EFA, CFA) assessed psychometric properties.

**Results:**

EFA revealed four factors: Satisfaction, Patient Safety, Care Pathway Safety, and Handover Content. CFA confirmed good model fit. Inter‐factor correlations showed strong links between handover quality and safety, alongside discrepancies between satisfaction and objective standards.

**Linking Evidence to Practice:**

The HAND‐Q offers a practical tool to assess handover quality, support standardization, enhance patient safety, and inform training and digital solutions.

## 1 Background

Nursing handover is a structured and essential process through which patient care responsibilities and critical information are transferred from one nurse to another, ensuring continuity and safety in care (Khalaf 2023). Effective handovers facilitate seamless transition of responsibility and accountability, minimizing the risk of errors and enhancing patient outcomes (Ghosh et al. 2021; Slade et al. 2018).

Various handover styles exist, differing between wards and shifts. These styles encompass different approaches, including bedside handover, which directly involve the patient, verbal, non‐verbal, or digital documentation‐based handovers (Bakon et al. 2017; Messam and Pettifer 2009; Tacchini‐Jacquier et al. 2020). When performed effectively, nursing handovers optimize patient safety, facilitate high‐quality care delivery, and improve overall healthcare efficiency.

Patients report that bedside handovers enhance their sense of safety, continuity, and participation in care, providing clearer information and greater reassurance during hospitalization (Bressan et al. 2019). At the organizational level, a ward‐based program combining training with process redesign reduced hospital‐acquired complications and strengthened the culture of patient‐centered care (Chien et al. 2022).

Moreover, structured communication during handovers enhances collaboration among healthcare providers and actively involves patients in their care. This approach has been associated with a reduction in falls (−9.3% to −80%) and medication errors (−11% to > −50%) although evidence for pressure injuries is mixed (Hada and Coyer 2021). Consistently, poor handover has been linked to omissions, delays, and treatment errors, whereas structured, patient‐involving practices enhance information transfer and satisfaction (Desmedt et al. 2021; Wang et al. 2022). In addition to these clinical outcomes, a recent systematic review reported efficiency gains; bedside handover lasted 83–204 s per patient, overtime decreased by ~10 min/day (~23% of the unit salary budget), and call‐light usage was reduced by one‐third (Daicampi et al. 2025).

Research indicates that nurses' perceptions of handover practices are pivotal; those who feel adequately supported and trained are more likely to conduct efficient handovers (Chien et al. 2022; Malfait et al. 2019). Evidence supports simulation‐based workshops, role play and the use of standardized protocols as effective educational strategies to strengthen communication skills (Hada and Coyer 2021). Sustained improvement, however, requires coupling training with organizational support—leadership endorsement, protected time, mentoring and redesigned tools—to embed patient‐centred communication and reduce complications (Chien et al. 2022; Desmedt et al. 2021).

Despite their critical role, nursing handovers are often compromised by interruptions, lack of standardization, and time constraints, leading to variability in practice and dissatisfaction among healthcare professionals (Raeisi et al. 2019).

During the European NEXT (Nursing Early Exit Study), satisfaction about the handover process has been assessed in 10 European countries (Meissner et al. 2007). The percentage of nurses dissatisfied with shift handovers varied significantly across the 10 countries. England, Poland, and Slovakia reported the lowest levels of dissatisfaction. In contrast, France recorded the highest dissatisfaction rate, with 61% of respondents expressing dissatisfaction. High levels of dissatisfaction were also observed in Italy and Germany. The most frequently declared cause for dissatisfaction with shift handovers was “too many disturbances” (Meissner et al. 2007). The disturbances can come from different sources, such as the patient's family (Tobiano et al. 2017) or other healthcare workers (Giske et al. 2018).

Previous studies have explored nurses' perception, satisfaction, and efficacy regarding handovers (Loefgren Vretare and Anderzén‐Carlsson 2020; Weston et al. 2022). These studies have allowed us to understand and identify negative attitudes and practices toward the process, but also the positive aspects. However, few studies have used a reliable and valid instrument.

Existing tools, such as the Handover Evaluation Scale (HES) (O'Connell et al. 2014), and the Handover Quality Rating Form (HQRF) (Manser et al. 2010) primarily assess information transfer and efficiency but fail to incorporate nurses' perceptions of handover quality and patient safety.

Given these limitations, there is a need for a comprehensive, psychometrically validated instrument that evaluates both handover quality and nurses' satisfaction. This study aims to fill this gap by developing and validating the Handover Quality Questionnaire (HAND‐Q), a new tool designed to assess key aspects of handover effectiveness, efficiency, and perceived safety.

## 2 Aim

The aim of this study was to develop and validate a new instrument for measuring nurses' satisfaction about handover method: the Handover Quality Questionnaire (HAND‐Q).

## 3 Methods

### 3.1 Design

This study adopted a methodological multi‐method and multi‐phase research design aimed at developing and validating a measurement tool for assessing nurses’ satisfaction with handover practices. According to the recommendations for scale design and development (Rattray and Jones 2007), the study adhered to the ‘STrengthening the Reporting of OBservational studies in Epidemiology’ (STROBE) checklist as well (Cuschieri 2019) as Enhancing the QUAlity and Transparency of Health Research’ (EQUATOR) guidelines (Simera et al. 2010).

### 3.2 Scale Development

The development of the Handover Quality Questionnaire was carried out in two main phases: the conceptualization phase and the validation phase.

#### 3.2.1 Conceptualization Phase

The conceptualization phase marked the initial step in the development of the HAND‐Q scale. This phase began with a literature review conducted in May 2023, aimed to identify key elements of quality of handover practices. Following this, a group discussion was organized involving the research team, nurse managers, and nurse educators. This discussion facilitated a comprehensive analysis of the literature review findings and allowed for the achievement of a shared consensus on the essential aspects to be included in the scale. The final step in this phase focused on drafting an initial pool of items, which were categorized into two sections: one addressing the description of the handover process and the other assessing its quality.

#### 3.2.2 Validation Phase

The validation phase built upon the work of the conceptualization phase and consisted of three stages. In the first stage, a pilot study was conducted with 83 nurses from various adult medical and surgical departments within a University Hospital. This stage aimed to evaluate the initial version of the HAND‐Q scale and ensure its face and content validity. Based on the results of the pilot study, the research team revisited and refined the scale during the second stage, leading to the reformulation of five items for improved clarity. The revised version of the HAND‐Q was then uploaded to the REDCap platform for further testing. In the final stage, the scale was administered to a larger sample of 514 nurses working in a University Hospital. This step included a thorough assessment of the factorial structure and reliability of the scale, ensuring its robustness and applicability.

### 3.3 Handover Quality Questionnaire (HAND‐Q)

The questionnaire was developed to assess the bedside handover process among nursing staff and was structured into three sections: demographic section, handover description, and handover quality questionnaire.

#### 3.3.1 Demographic Section

The demographic section (5 items), designed to gather information on participants' unit of assignment, age, gender, educational qualifications, years of professional experience, and current role within their unit.

#### 3.3.2 Handover Description Section

The handover description section (11 items), focusing on the operational aspects of the handover process including key actors involved in handovers, locations where handovers occurred, communication methods (e.g., verbal exchanges, informal notes, formal documentation, or digital tools), and the number of patients typically discussed. This section also explored the perceived optimal handover durations and disruptive factors (e.g., interruptions from colleagues, patients, or caregivers).

#### 3.3.3 Handover Quality Questionnaire

The Handover Quality Questionnaire (19 items) evaluated handover effectiveness, efficiency, and quality, focusing on participants' satisfaction with the handover, the consistency and relevance of the information shared, and the extent to which the process ensured safe and continuous care. Additionally, this section explored the occurrence of errors, delays, and misunderstandings during handovers, as well as the impact of interruptions on the quality of the process. Participants responded to the items using a 4‐point Likert scale. Specifically, items 3.1–3.4 assessed agreement ranging from 1 (strongly disagree)to 4 (strongly agree); items 3.10 and 3.11 evaluated frequency, from 1 (never) to 4 (always), with intermediate options of rarely and almost always; all other items similarly measured frequency, with response options ranging from 1 (never) to 4 (always), including intermediate options of sometimes and often.

### 3.4 Statistical Methods

Descriptive statistics were used for the first two subscales of the questionnaire: Demographic section and Handover description section categorical variables expressed as absolute frequencies and percentages, and continuous variables summarized using means, standard deviations (SD), medians, and the minimum and maximum values. Although Likert‐type items are ordinal by nature, scales with four or more response options are often treated as approximately interval‐level data in health and social sciences research. This approach allows the mean to represent central tendency and the standard deviation to capture variability, providing more informative summaries and enabling easier comparison across items and groups than the median and interquartile range.

To examine the psychometric properties of the HAND‐Q, a two‐step validation approach was undertaken, consisting of Exploratory Factor Analysis (EFA), followed by Confirmatory Factor Analysis (CFA). The analyses were conducted using IBM SPSS Statistics version 29.0 (SPSS Inc., Chicago, IL, USA), except for CFA, which was performed using MPlus software version 7. The adequacy of the sample was ensured by including 514 nurses, a number largely exceeding the recommended ratio of 5 to 10 participants per item, thereby providing sufficient statistical power for factor analysis (Hair et al. 2010).

EFA was performed to explore the latent dimensionality of the observed variables. To assess the dataset's suitability for factor analysis, the Kaiser‐Meyer‐Olkin (KMO) measure of sampling adequacy (Kaiser 1974) and Bartlett's test of sphericity (Bartlett 1954) were utilized. A KMO value greater than 0.6 was considered acceptable, confirming the adequacy of sampling. Bartlett's test determines if the correlations among variables are sufficient for factor extraction, with a significance level set at *p* < 0.05 (Wu et al. 2023).

Communality value (h^2^) was also evaluated with a threshold of 0.20 adopted as the minimum acceptable level (Child 1975). Items with communalities below this value were considered poorly represented by the extracted factors and thus candidates for removal or revision.

For factor extraction, Principal Component Analysis (PCA) was used, as it effectively uncovers the underlying structure of observed variables. The Pattern Matrix, derived from varimax rotation, was employed to interpret the factor structure (Kaiser 1958). This matrix displays the factor loadings, which depict the strength of the connection between each observed variable and the latent factors. Factor loadings with 𝑥 ≥ 0.3 were deemed minimally acceptable for a factor inclusion, whereas higher values (e.g., > 0.4 or > 0.5) were regarded as indicating stronger and more meaningful relationships (Wu et al. 2023). Variables with cross‐loadings or ambiguous loadings were meticulously assessed based on theoretical criteria to ensure the factor structure's validity.

In the second step a CFA was carried out to validate the factor structure suggested by the EFA. The CFA was planned to be conducted using the Maximum Likelihood Robust (MLR) estimation method. Model fit was assessed using multiple indices, including the chi‐square statistic, the Root Mean Square Error of Approximation (RMSEA), the Comparative Fit Index (CFI), and the Standardized Root Mean Square Residual (SRMR). According to Hu and Bentler (1999), acceptable thresholds were to be considered (e.g., RMSEA below 0.06, CFI values above 0.90, and SRMR below 0.08). Finally, the explained variance (indicator reliability, *R* ^2^) was examined to assess the contribution of each item to its latent factor. An *R* ^2^ value ≥ 0.30 was considered acceptable, with higher values indicating stronger explanatory power (Hair et al. 2010).

This comprehensive analytical approach, supported by the methodological frameworks proposed by Kline (2011) and Byrne (2010), was expected to yield a robust measurement model for assessing handover quality.

### 3.5 Ethical Considerations

This study was carried out according to the criteria set by the Declaration of Helsinki and the protection of personal data was ensured in accordance with Regulation 2016/679 of the European Parliament. The research project received approval from the Territorial Ethics Committee Area Centro‐Est Veneto (Prot. 0033356/25, internal code CET‐ACEV: 548n/25).

## 4 Results

### 4.1 Description of the Sample

A total of 514 nurses participated in the study (Table 1). The majority were female (86.6%), with a mean age of the sample 42.26 years (SD = 12.04). Regarding education level, most participants held a bachelor's degree (76%), followed by those with a first‐level postgraduate qualification (21.6%). A smaller proportion had completed a master's degree (1.9%) or a second‐level postgraduate qualification (0.4%). The distribution of work areas showed a predominance of professionals in the medical (27%) and surgical fields (26.3%), followed by those in maternal and childcare (18.5%), critical care (15.8%), and neonatal/pediatric care (12.5%). Professional experience varied: 2.9% had less than one year of experience, 18.1% had worked for 1–5 years, and 17.9% for 5–10 years. The majority, however, had over 10 years of experience (61.1%). Most participants worked as shift nurses (72.4%).

**TABLE 1:** Demographic characteristics (*N* = 514).

Characteristics	
Age	
Mean (SD)	42.26 (12.04)
Median (min–max)	43 (23–66)
Area of work, *N* (%)	
Medical area	139 (27%)
Surgical area	135 (26.3)
Maternal and child area	95 (18.5)
Critical care area	81 (15.8)
Neonatal/pediatric area	64 (12.5)
Gender, *N* (%)	
Male	65 (12.6)
Female	445 (86.6)
Not specified	4 (0.8)
Education level, *N* (%)	
Bachelor's degree	391 (76)
First‐level post Bachelor's Degree	111 (21.6)
Master's degree	10 (1.9)
Second‐level post Master's Degree	2 (0.4)
Years in Profession, *N* (%)	
< 1 year	15 (2.9)
1–5 years	93 (18.1)
5–10 years	92 (17.9)
> 10 years	314 (61.1)
Role in organization	
Day Nurse	113 (22)
Shift Nurse	372 (72.4)
Head nurse	29 (5.6)

Preliminary subgroup analyses did not reveal substantial differences in HAND‐Q scores based on age, years of professional experience, or working area.

### 4.2 Characteristics of Handover

The characteristics of handover are presented in Table 2. Handover practices revealed that the main participants were the outgoing and incoming nurses (*N* = 502), supported by other nurses involved in the handover (*N* = 104), support staff (*N* = 79), head nurses (*N* = 29), physicians (*N* = 36), and nursing students (*N* = 31). During handovers, other nurses performing procedures were often present (*N* = 147), along with caregivers or family members (*N* = 15). Handovers predominantly took place in the nursing office (*N* = 282; 54.7%) or the unit breakroom (*N* = 110; 21.4%), with fewer occurring at the patient's bedside (*N* = 96; 18.7%).

**TABLE 2:** Handover description.

Characteristics	
Main Participants^a^, *N* = 781 (%)	
Outgoing/incoming nurse	502 (64.3)
Other nurses involved in the handover	104 (13.3)
Support staff	79 (10.1)
Head nurse	29 (3.7)
Physicians	36 (4.6)
Nursing students	31 (4)
Subject Present During the Handover^a^, *N* = 901 (%)	
Other Nurses Involved in the Handover	378 (42)
Support staff	168 (18.7)
Other nurses performing procedures	147 (16.3)
Nursing students	131 (14.5)
Head nurse	39 (4.3)
Physicians	23 (2.5)
Caregivers/family members	15 (1.7)
Handover mainly occur place. *N* = 514 (%)	
Nursing office	281 (54.7)
Unit breakroom	110 (21.4)
At the patient's bedside	96 (18.7)
Hallway	23 (4.59)
Kitchen	3 (0.6)
Mode of handover conducted^a^, *N* = 514 (%)	
Verbal only	215 (41.8)
Written using unit‐specific forms	123 (23.9)
Written informal notes	98 (19.1)
Written using an electronic medical record	78 (15.2)
Number of patients involved in the handover. *N* = 514 (%)	
11–15 patients	216 (42)
1–5 patients	147 (28.6)
6–10 patients	96 (18.7)
16–20 patients	55 (10.7)
Minutes adequate for handover. *N* = 514 (%)	
10 min	75 (14.6)
15 min	231 (44.9)
20 min	147 (28.6)
25 min	30 (5.8)
30 min	27 (5.3)
More than 30 min	4 (0.8)
Factors disrupt the handover^a^, *N* = 1107 (%)	
Ringbell or patient/caregiver Interruptions	347 (31.3)
Physicians	304 (27.5)
Telephone calls	255 (23)
Other nurses	201 (18.2)
Essential information about patients at the start of a shift^a^ *N* = 1768 (%)	
Significant Changes in Patient Condition	416 (23.5)
Care priorities	255 (14.4)
Psychophysical status	180 (10.2)
Vital sign status	155 (8.8)
Wound/dressing condition	114 (6.4)
Accesses/devices	113 (6.4)
Therapeutic plan	105 (5.9)
Current therapy	95 (5.4)
Mobility	79 (4.5)
Medical history	73 (4.1)
Procedures already performed	72 (4.1)
Discharge plan	46 (2.6)
Monitoring frequency	37 (2.1)
Possible prognosis	28 (1.6)
Participation in handover training courses. *N* = 514 (%)	
Never	365 (71)
More than 5 years ago	90 (17.5)
2–5 years ago	42 (8.2)
1–2 years ago	10 (1.9)
In the last year	7 (1.4)
Awareness of tools to make the handover more effective. *N* = 514 (%)	
Aware and I try/would like to use them	188 (36.6)
Vaguely aware	153 (29.8)
Never heard of them	117 (22.8)
Aware. but unsuitable for my context	39 (7.6)
Aware. but no time to use them	17 (3.3)
Knowledge of handover tools^a^ *N* = 495 (%)	
SBAR	384 (77.6)
SAMPLE	56 (11.3)
ISOBAR	28 (5.6)
MIST	27 (5.5)

Abbreviations: ISOBAR, identify, situation, observation, background, agree a plan, readback; MIST, mechanism, injury pattern, sign, treatment; SAMPLE, signs and symptoms, age, athleticism, allergies, medication, past history, last oral intake, events leading up to incident; SBAR, situation, background, assessment, recommendation.

^a^ Interviews can select all that apply.

The handover process was primarily conducted verbally only (*N* = 215; 41.8%), unit‐specific forms (*N* = 123; 23.9%), followed by informal written notes (*N* = 98; 19.1%) or electronic medical records (*N* = 78; 15.2%). As reported by the nurses, the most common duration for handovers was 15 min (*N* = 231; 44.9%), followed by 20 min (*N* = 147; 28.6%), while longer durations were less frequent.

Several factors disrupted the handover process, such as interruptions from patients or caregivers (*N* = 347), colleagues (*N* = 201), physicians (*N* = 304), and telephone calls (*N* = 255). The most frequently communicated information during handovers included significant changes in the patient's condition (*N* = 416), care priorities (*N* = 255), psychophysical status (*N* = 180), and wound/dressing conditions (*N* = 114). Less frequently reported items included patient mobility or discharge plans.

Only a small proportion of participants had attended handover training courses in the past five years (*N* = 90; 17.5%), while 71% (*N* = 365) had never received specific training. Furthermore, 36.6% (*N* = 188) of participants were aware of tools to improve handover practices and either used them or expressed interest in doing so. Among these tools, Situation Background Assessment Recommendation—SBAR—was the most well‐known (*N* = 384), followed by Signs and symptoms, Age, athleticism, allergies, Medication, Past history, Last oral intake, Events leading up to incident—SAMPLE (*N* = 56), Identify, Situation, Observation, Background, Agree a Plan, Readback—ISOBAR – (*N* = 28), and Mechanism, Injury Pattern, Sign, Treatment—MIST (*N* = 27).

### 4.3 Handover Quality Questionnaire

The HAND‐Q questionnaire consists of 19 items rated on a four‐point scale. The first four items (3.1–3.4) assess the level of agreement with statements regarding satisfaction, effectiveness, and efficiency of the handover process, while the remaining items evaluate the frequency of specific occurrences, ranging from “never” to “always.” Overall, the items capture four core domains: nurses' satisfaction with handovers, perceived patient safety, care pathway safety, and the completeness and relevance of handover content.

Descriptive results (Table 3) indicated only moderate levels of satisfaction, effectiveness, and efficiency (*M* values between 2.7 and 2.9), whereas severe adverse events such as medication errors or patient harm were reported as infrequent (*M* values between 1.1 and 1.7). In contrast, two critical issues emerged clearly: frequent interruptions during handovers (*M* = 3.18) and considerable variability in the way individual nurses conduct the process (*M* = 3.01). These findings highlight factors that undermine the standardization and smooth flow of information transfer.

**TABLE 3:** Description of the handover quality questionnaire (*N* = 514).

Item	Mean (SD)
3.1 Are you satisfied with the current nursing handovers?	2.74 (0.68)
3.2 Is the current handover method effective?	2.87 (0.6)
3.3 Is the current handover method efficient?	2.74 (0.68)
3.4 Each nurse has their own way of giving handovers	3.01 (0.74)
3.5 Medication errors	1.35 (0.53)
3.6 Harm/injury to the patient	1.11 (0.32)
3.7 Failure to deliver nursing care	1.75 (0.54)
3.8 Delay in care delivery	1.85 (0.6)
3.9 Miscommunication with other healthcare professionals	2.06 (0.56)
3.10 Is the time allocated for handovers sufficient?	2.53 (0.7)
3.11 Are there interruptions during handovers?	3.18 (0.76)
3.12 They ensure safe and adequate care	3.2 (0.49)
3.13 They include the necessary information to set care priorities	3.27 (0.55)
3.14 They provide a complete overview of the patient's clinical situation	3.08 (0.62)
3.15 The handover content ensures continuity of care	3.27 (0.57)
3.16 The nurse receiving the handover is interested	3.38 (0.57)
3.17 There is a discrepancy between verbal and written notes	1.98 (0.59)
3.18 The nurse giving the handover includes irrelevant information	2.24 (0.66)
3.19 The nurse giving the handover omits important information	2.01 (0.57)

*Note:* Mean = average response; SD = standard deviation, indicating response variability. For items 3.1–3.4 response options were 1 (strongly disagree), 2 (disagree), 3 (somewhat agree) and 4 (strongly agree). For items 3.10 and 3.11 response options were 1 (never), 2 (rarely), 3 (almost always) and 4 (always). For all other items response options were 1 (never), 2 (sometimes), 3 (often) and 4 (always).

Regarding content quality, items related to clinical safety, and the comprehensiveness of information received relatively high mean scores (*M* between 3.1 and 3.3), suggesting that handovers generally succeed in providing the essential data required for continuity of care. Nonetheless, other aspects revealed notable weaknesses, such as the perception that allocated time for handovers is insufficient (*M* = 2.53), the frequency of interruptions (item 3.11), and inconsistencies between verbal and written notes (item 3.17).

In the EFA, the Kaiser–Meyer–Olkin measure of sampling adequacy was 0.878, and Bartlett's test of sphericity indicated that the correlation matrix was not random, χ^2^(171) = 2719.339, *p* < 0.001. Factor extraction was performed using principal axis factoring, followed by Varimax rotation with Kaiser normalization, which converged in six iterations. The factorial structure of the Handover Quality Questionnaire consisted of four factors: (1) Satisfaction (5 items, 28.6% of cumulative variance) with factor loadings ranging from 0.575 to 0.759; (2) Patient Safety (5 items, 38.6% of cumulative variance) with factor loadings ranging from 0.523 to 0.679; (3) Care pathway safety (5 items, 46.1% of cumulative variance) with factor loadings ranging from 0.511 to 0.844, and (4) Handover content (4 items, 52.2% of cumulative variance) with factor loadings ranging from 0.514 to 0.664 (Table 4).

**TABLE 4:** Exploratory factor analysis of the handover quality questionnaire (*N* = 514).

Items	*h* ^2^	Satisfaction	Patient safety	Care pathway safety	Handover content
3.1 Are you satisfied with the current nursing handovers?	0.616	**0.711**	−0.150	0.234	−0.181
3.2 Is the current handover method effective?	0.547	**0.610**	−0.248	0.323	−0.093
3.3 Is the current handover method efficient?	0.654	**0.749**	−0.162	0.257	−0.041
3.4 Each nurse has their own way of giving handovers.	0.286	−0.058	0.044	−0.128	**0.514**
3.5 Medication errors	0.524	−0.042	**0.672**	−0.185	0.188
3.6 Harm/injury to the patient	0.515	−0.017	**0.697**	−0.122	−0.118
3.7 Failure to deliver nursing care	0.540	−0.216	**0.651**	−0.106	0.241
3.8 Delay in care delivery	0.477	−0.373	**0.523**	0.012	0.253
3.9 Miscommunication with other healthcare professionals	0.519	−0.297	**0.549**	−0.085	0.349
3.10 Is the time allocated for handovers sufficient?	0.335	**0.575**	−0.065	−0.004	0.034
3.11 Are there interruptions during handovers?	0.393	**−0.595**	0.019	0.008	0.196
3.12 They ensure safe and adequate care	0.609	0.032	−0.178	**0.756**	−0.076
3.13 They include the necessary information to set care priorities	0.746	0.128	−0.095	**0.844**	−0.092
3.14 They provide a complete overview of the patient's clinical situation	0.620	0.193	−0.137	**0.747**	−0.077
3.15 The handover content ensures continuity of care	0.705	0.127	−0.090	**0.814**	−0.132
3.16 The nurse receiving the handover is interested	0.419	0.120	−0.001	**0.511**	−0.379
3.17 There is a discrepancy between verbal and written notes	0.456	−0.037	0.150	−0.190	**0.629**
3.18 The nurse giving the handover includes irrelevant information	0.468	−0.141	0.064	0.057	**0.664**
3.19 The nurse giving the handover omits important information	0.495	−0.085	0.243	−0.175	**0.631**

*Note:* *h* ^2^ communality. Communality values > 0.70 were considered well explained, while values < 0.20 were considered poorly represented by the extracted factors and thus candidates for removal or revision. Bold numbers indicate the highest loading of each item on its corresponding factor after rotation.

To further validate the factorial structure, CFA was conducted using the Maximum Likelihood Robust (MLR) estimation method (Supplementary 1). The model fit indices indicated that the four‐factor model provided an adequate representation of the data. Statistics fit indices suggested good model adequacy, with an RMSEA of 0.040 (90% CI: 0.032–0.048), a CFI of 0.951, and an SRMR of 0.044. These values suggest that the proposed structure aligns well with the observed data.

Factor loadings were generally strong (Table 5), supporting the construct validity of the instrument. However, some items displayed lower loadings, such as item 3.10 ‘Is the time allocated for handovers sufficient?’ (0.343) and 3.11 ‘Are there interruptions during handovers?’ (−0.402) in Satisfaction and 3.4 ‘Each nurse has their own way of giving handovers’ (0.324) in Handover. Notably, item 3.11 was a reverse‐scored item, which could have influenced its loading by introducing response pattern differences compared to the other items within the Satisfaction factor. Similarly, some items explained lower variance (e.g., item 3.6 “Harm/injury to the patient”), suggesting weaker alignment with their intended factors. Although these items were not removed at this stage, their performance indicates that they may require revision or further testing in future validations, as they still reflect relevant aspects of real‐world handover practice.

**TABLE 5:** Confirmatory factor analysis of the HAND‐Q (*N* = 514).

Items	Factor loadings		*r* ^2^	
	Estimate	se	Estimate	se
Satisfaction				
3.1 Are you satisfied with the current nursing handovers?	0.764	0.033	0.0584	0.051
3.2 Is the current handover method effective?	0.769	0.031	0.091	0.047
3.3 Is the current handover method efficient?	0.723	0.032	0.523	0.046
3.10 Is the time allocated for handovers sufficient?	0.343	0.047	0.118	0.033
3.11 Are there interruptions during handovers?	−0.402	0.040	0.162	0.032
Patient safety				
3.5 Medication errors	0.557	0.039	0.310	0.044
3.6 Harm/injury to the patient	0.372	0.046	0.139	0.034
3.7 Failure to deliver nursing care	0.693	0.036	0.480	0.050
3.8 Delay in care delivery	0.656	0.041	0.431	0.053
3.9 Miscommunication with other healthcare professionals	0.702	0.031	0.493	0.044
Care pathway safety				
3.12 They ensure safe and adequate care	0.721	0.031	0.520	0.044
3.13 They include the necessary information to set care priorities	0.833	0.024	0.695	0.040
3.14 They provide a complete overview of the patient's clinical situation	0.724	0.025	0.524	0.037
3.15 The handover content ensures continuity of care	0.807	0.025	0.651	0.041
3.16 The nurse receiving the handover is interested	0.488	0.040	0.238	0.039
**Handover content**				
3.4 Each nurse has their own way of giving handovers.	0.324	0.059	0.105	0.038
3.17 There is a discrepancy between verbal and written notes	0.573	0.055	0.328	0.063
3.18 The nurse giving the handover includes irrelevant information	0.469	0.056	0.220	0.053
3.19 The nurse giving the handover omits important information	0.674	0.055	0.454	0.075

Abbreviation: SE, Standard error.

Descriptive results (Table 3) further contextualize these findings. Nurses reported only moderate levels of satisfaction, effectiveness, and efficiency (mean values between 2.7 and 2.9). In contrast, severe adverse events such as medication errors or patient harm were reported as rare (mean values between 1.1 and 1.7). The most critical issues concerned the frequency of interruptions (*M* = 3.18) and variability in individual handover practices (*M* = 3.01), both of which can compromise standardization and the overall quality of the process. Items addressing the adequacy and completeness of information (items 3.12–3.15) scored relatively higher (*M* between 3.1 and 3.3), suggesting that essential patient data were generally conveyed. However, other aspects such as insufficient time allocation (item 3.10, *M* = 2.53) and discrepancies between verbal and written notes (item 3.17, *M* = 1.98) emerged as weak points, indicating organizational and environmental rather than informational barriers.

Inter‐factor correlations provided additional insights into the relationships between constructs. A strong negative correlation was found between Satisfaction and Patient Safety (*r* = −0.650; *p* < 0.001), suggesting potential conceptual overlap or measurement inconsistencies. Satisfaction and Care Pathway Safety were positively correlated (*r* = 0.515; *p* < 0.001), indicating a moderate relationship between these dimensions. Satisfaction and Handover Content exhibited a negative correlation, while Patient Safety and Handover Content were positively associated (*r* = 0.662; *p* < 0.001). Finally, Care Pathway Safety and Handover Content had a moderate negative correlation (*r* = 0.447; *p* < 0.001), suggesting interdependencies among these factors.

The negative correlation between Satisfaction and Handover Content may indicate a tension between perceived completeness and subjective satisfaction. More detailed or structured handovers, while ensuring comprehensive information transfer, may be perceived as time‐consuming or burdensome, leading to lower satisfaction. Conversely, shorter or less detailed handovers may be perceived as more satisfactory but could compromise content quality. This finding highlights the challenge of balancing efficiency and completeness in clinical communication, and underlines the importance of adopting structured, standardized tools that facilitate both clarity and brevity.

The explained variance analysis indicated that certain items demonstrated strong contributions to their respective factors, while others exhibited lower variance explanations. Items with high explained variance (*R* ^2^ > 0.6) included Item 3.13 ‘They include the necessary information to set care priorities’ (*R* ^2^ = 0.695) and Item 3.15 ‘The handover content ensures continuity of care’ (*R* ^2^ = 0.651), suggesting strong associations with their latent constructs. Conversely, some items showed lower variance explanation (*R* ^2^ < 0.3), including Item 3.4 ‘Each nurse has their own way of giving handovers’ (*R* ^2^ = 0.105), Item 3.10 ‘Is the time allocated for handovers sufficient?’ (*R* ^2^ = 0.118), and Item 3.6 ‘Harm/injury to the patient’ (*R* ^2^ = 0.139), which may indicate weaker alignment with their intended factors and highlight the need for further refinement.

## 5 Discussion

The objective of this study was to develop and validate a new instrument for measuring nurses' satisfaction with the handover process: the Handover Quality Questionnaire (HAND‐Q). The need for such an instrument arises from the lack of validated tools that comprehensively assess both handover quality and nurses' perceptions of the process's safety.

Although demographic characteristics were not the primary focus of this study, some noteworthy patterns emerged. Nurses with greater professional experience and those employed in critical care units tended to report higher perceptions of patient safety and continuity during handovers. This may be explained by their greater familiarity with structured communication protocols and their frequent participation in multidisciplinary teamwork, which are common in high‐acuity settings. In contrast, younger or less experienced nurses appeared to perceive handovers as more fragmented, likely reflecting limited exposure to standardized approaches. These differences suggest that demographic and professional factors may influence perceptions of handover quality, underscoring the need for future studies to examine such variations more systematically. This finding also implies that targeted training could help reduce this gap (Yusrawati et al. 2022).

The data also revealed that handovers were primarily conducted verbally, with limited use of written tools or electronic medical records. However, literature suggests that relying solely on verbal communication is inadequate and prone to significant data loss. While careful note‐taking during handover improves information retention, the use of a standardized pre‐printed sheet containing key patient details has been shown to nearly eliminate data loss (Bhabra et al. 2007). Moreover, the implementation of structured approaches, such as checklists, has been widely recognized for enhancing communication consistency among healthcare providers. Checklists have been effectively used to reduce morbidity and mortality in both medical and surgical settings (Methangkool et al. 2019), and are also associated with improved nursing satisfaction and handover efficiency (Caruso et al. 2015).

In our study context, the reliance on verbal exchanges may reflect cultural habits or lack of institutional investment in written or electronic systems. From a practical standpoint, promoting the use of standardized templates or integrating electronic documentation into routine practice could reduce variability and prevent information loss. The handover process mainly took place in the nursing office or the breakroom, with a lower percentage occurring at the patient's bedside. While literature indicates that bedside handover enhances the exchange of crucial information between nurses and patients, reducing the risk of miscommunication (Mardis et al. 2016; Tobiano et al. 2017), its implementation remains limited. Patients strongly prefer bedside handover, valuing the opportunity to be involved in their care. However, nurses, while recognizing patient participation as a key aspect, generally prefer conducting handover away from the bedside when other factors are equal (Oxelmark et al. 2020). These contrasting perspectives highlight the need to balance patient‐centered communication with practical and logistical considerations in clinical practice.

Frequent interruptions, primarily from patients, caregivers, physicians, and phone calls, were identified as a major challenge. This finding is consistent with existing literature, which identifies interruptions as a key factor compromising patient safety and continuity of care. Distractions have been recognized as a major contributor to handover failure, leading to incomplete transfer of information and the omission of important tasks (Methangkool et al. 2019). Poor‐quality handover communication can have serious consequences for patients, including lack of follow‐up on treatments and medications, inefficient task management, and poor coordination of time and space (Ghosh et al. 2021; Raeisi et al. 2019). Additionally, Raeisi et al. (2019) highlight that one of the most significant challenges in handover quality is the communication and relationship between incoming and outgoing nurses, further emphasizing the need for structured and standardized handover practices to mitigate these risks (Raeisi et al. 2019). The identification of disruptive factors during handovers, such as interruptions from colleagues, patients, patients, or caregivers, is consistent with studies that highlight the negative impact of environmental distractions on handover efficiency (Giske et al. 2018; Tobiano et al. 2017).

Our findings also showed that interruptions from patients and family members were commonly reported, even though most handovers occurred in the nursing office rather than at the bedside. This suggests that requests for information or assistance frequently overlap with the time dedicated to shift changes, and that patients or caregivers may perceive this moment as an opportunity to approach nurses regardless of where the handover is conducted. Previous studies confirm that bedside handovers, while improving information accuracy and patient engagement, are associated with a higher risk of interruptions (Tobiano et al. 2017). In our context, this may be particularly relevant given the lack of protected spaces and formal organizational policies safeguarding handover time. These environmental and cultural aspects may explain why interruptions emerged so prominently in our data. From a practical perspective, this finding highlights the importance of implementing “protected handover” policies, such as limiting access to the nursing office during shift changes or introducing clear signage to reduce interruptions. Future research should evaluate whether these strategies can reduce communication errors and improve continuity of care.

Regarding the knowledge of handover tools, the majority of nurses were familiar with the SBAR (Situation, Background, Assessment, Recommendation) method. This aligns with findings from a systematic review on nursing handovers, which identified SBAR as the most frequently used handoff tool (Uhm et al. 2018).

Moreover, the low percentage of nurses receiving formal handover training (17.5%) highlights a critical gap in professional development. Literature suggests that nurses who receive structured training on patient handover demonstrate significantly better communication and collaboration skills. In the study by Karakurt et al. (2023), nurses who underwent handover training scored higher on the Patient Handover Evaluation Scale (PHES), particularly in the interaction and support sub‐dimension, compared to those without training (Karakurt et al. 2023). This finding aligns with broader evidence indicating that training programs are among the most effective solutions to improve shift handover processes, enhancing nurses' knowledge and confidence in patient handover. Similarly, Olasoji et al. (2019) found that targeted training for mental health nurses significantly improved the quality of shift handovers. In our sample, the scarcity of training may be related to the absence of mandatory institutional policies and the reliance on informal peer learning.

The results of exploratory and confirmatory factor analyses supported the validity of the four‐factor structure of the Handover Quality Questionnaire (HAND‐Q), a novel instrument designed to assess the quality and efficiency of nursing handovers from the nurses' perspective. These analyses confirmed a four‐factor structure comprising Satisfaction, Patient Safety, Care Pathway Safety, and Handover Content. The HAND‐Q demonstrated acceptable‐to‐good reliability for three of these factors, with the overall validation indicating a good model fit to the collected data, as evidenced by adequate model fit indices. However, the Satisfaction factor exhibited lower internal reliability, suggesting the need for further refinement to enhance its internal consistency.

The findings underscore the significant impact of structured handover practices on nurses' perceptions of safety and care continuity. Notably, the strong correlation between Handover Content and Patient Safety highlights the crucial role of clear and consistent information exchange in ensuring high‐quality transitions between shifts. Conversely, the negative correlation between Satisfaction and Patient Safety may indicate that nurses' subjective satisfaction with handover procedures does not always align with objective safety standards.

These results are consistent with previous research emphasizing the importance of structured communication during shift handovers (Bressan et al. 2019; Chien et al. 2022). Existing tools, such as the Handover Evaluation Scale (HES) (O'Connell et al. 2014) and the Handover Quality Rating Form (HQRF) (Manser et al. 2010), primarily focus on information transfer and efficiency. However, unlike these instruments, the HAND‐Q incorporates nurses' satisfaction and perceptions of patient safety, providing a more comprehensive assessment of the handover process. In this way, the HAND‐Q can serve not only as a research tool but also as a practical benchmark for healthcare institutions to identify weaknesses, inform training programs, and guide policy decisions aimed at improving both safety and satisfaction in handover practices.

### 5.1 Implications for Future Research

The HAND‐Q's robust psychometric properties and large sample provide a strong foundation for further research on handover quality. Nonetheless, reliance on self‐reported data may induce bias, as responses can be affected by social desirability, workplace culture, or expectations about ideal handovers. Future studies should therefore incorporate direct observational methods—such as structured audits, shadowing, or error tracking systems—to triangulate findings and reduce subjectivity. In addition, handover practices are shaped by institutional routines and cultural contexts, which may explain variations across settings. Multi‐site and cross‐cultural validation studies are needed to establish the generalizability of the HAND‐Q and to identify context‐specific determinants of handover quality. Future research should also explore links between HAND‐Q scores and patient outcomes, and assess the effectiveness of training interventions (e.g., simulation‐based workshops, standardized protocols) in improving both perceptions and observed practice. These efforts will not only advance methodological rigor but also generate evidence to inform policy recommendations and clinical guidelines.

### 5.2 Linking Evidence to Action

- The HAND‐Q was developed and validated, highlighting the need for structured handovers, standardized communication, and formal training to improve safety and efficiency.
- Hospitals and healthcare institutions should consider implementing evidence‐based handover models to improve consistency and reduce the risk of information loss.
- The HAND‐Q could serve as a benchmarking tool to evaluate handover quality across different units and hospitals.
- By systematically assessing nurses' perceptions, hospital administrators can identify areas for improvement and develop targeted interventions to optimize handover practices.

## 6 Conclusion

This study developed and validated the HAND‐Q, a novel instrument designed to assess nursing handover quality and satisfaction. Factor analysis confirmed a four‐factor structure—Satisfaction, Patient Safety, Care Pathway Safety, and Handover Content—demonstrating good reliability and validity.

Findings highlight the critical role of structured handover practices in ensuring patient safety and continuity of care. However, frequent interruptions, reliance on verbal communication, and lack of structured handover training remain key challenges that require targeted interventions.

## Funding

The authors have nothing to report.

## Conflicts of Interest

The authors declare no conflicts of interest.

## Supporting information


**Data S1:** wvn70098‐sup‐0001‐DataS1.docx.

## Data Availability

The data that support the findings of this study are available from the corresponding author upon reasonable request.
