# Supplementary material for: Nurses' Handover Satisfaction: Development and Validation of the Handover Quality Questionnaire
Source: Worldviews Evid Based Nurs. 2025 Dec 28;22(6):e70098. doi: 10.1111/wvn.70098 (PMC12745061; doi:10.1111/wvn.70098)
Supplement: Supplementary file 1 — Data S1: wvn70098‐sup‐0001‐DataS1.docx. [file WVN-22-0-s001.docx]

Supplementary Material 1. CFA model with standardized estimates (N = 514)


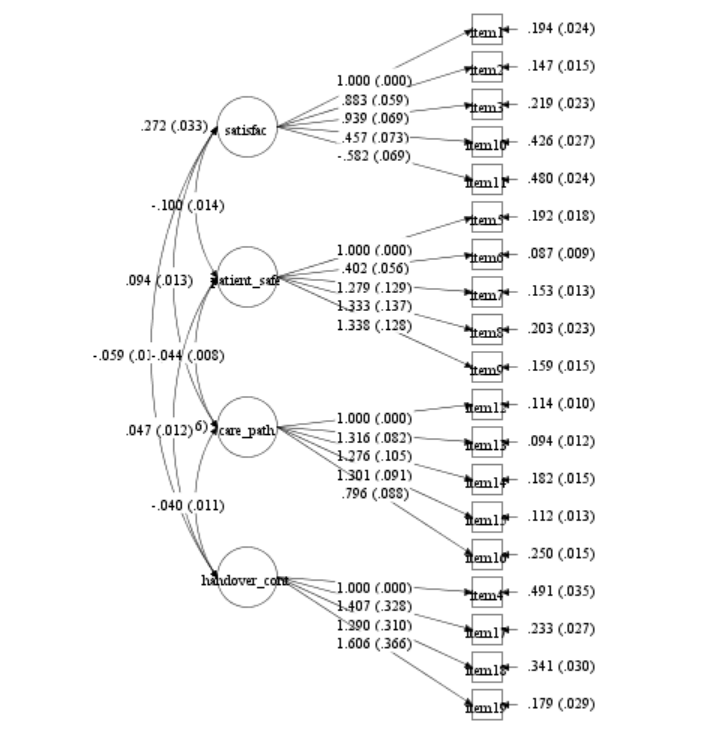


Supplementary Material 2. Handover Quality Questionnaire (HAND-Q)

1. **Demographic Section**

1.1 Unit of Affiliation: ______________________________________

1.2 Age

1.3 Gender

□ Male

□ Famale

□ Not declared

1.4 Highest Degree Earned (Select all that apply):

□ Diploma / Associate Degree

□ First-level Master's Degree

□ Bachelor’s Degree

□ Second-level Master’s Degree

□ PhD

1.5 How many years have you been practicing nursing?

□ Less than a year

□ 1 to 5 years

□ 5 to 10 years

□ Over 10 years

1.6 Role in the Unit Organization (Mark only one):

□ Shift Nurse

□ Day Nurse

□ Nurse Coordinator

**2. Description of Handover**

2.1 Who are the main participants in handovers? (Select all that apply):

□ Outgoing Shift Nurse / Incoming Shift Nurse

□ Other nurses exchanging handovers

□ Support staff (OSS, OTAA, ...)

□ Nurse Coordinator

□ Doctors

□ Nursing Students

2.2 Who is present during handover exchanges? (Select all that apply):

□ Other nurses performing procedures

□ Other nurses exchanging handovers

□ Support staff

□ Nurse Coordinator

□ Doctors

□ Nursing Students

□ Family members / caregivers

2.3 Where does the handover exchange primarily take place? (Select all that apply):

□ Nurse’s office

□ Unit’s station

□ Corridor

□ Kitchen

□ Patient’s bedside

2.4 How are handovers exchanged? (Select all that apply):

□ Verbally

□ In writing using informal notes

□ In writing using forms adopted in the Unit

□ In writing via the electronic medical record

2.5 How many patients do you handle during handovers?

□ 1 to 5 patients

□ 5 to 10 patients

□ 11 to 15 patients

□ 16 to 20 patients

2.6 How many minutes do you think are sufficient for an adequate handover for the entire sector/module?

□ 10 minutes

□ 15 minutes

□ 20 minutes

□ 25 minutes

□ 30 minutes

□ Over 30 minutes

2.7 What are the most disruptive factors during handovers? (Select all that apply):

□ Call bells or direct interruptions from patients / caregivers

□ Interruptions by other nurses

□ Interruptions by doctors

□ Phone calls

2.8 What are the essential pieces of patient information to know at the start of the shift? (Select all that apply):

□ Care priorities

□ Therapeutic plan

□ Current therapy

□ Significant changes in patient condition

□ Medical history

□ Psycho-physical state

□ Procedures already performed

□ Possible prognosis

□ Vital sign status

□ Monitoring frequency

□ Wound/dressing conditions

□ Access devices / medical tools

□ Mobilization

□ Discharge plan

2.9 Have you attended training courses on the handover process?

□ In the last year

□ 1 to 2 years ago

□ 2 to 5 years ago

□ Over 5 years ago

□ Never

2.10 Are you aware of tools to make handover more effective?

□ I have never heard of them

□ I’ve heard of them superficially

□ I know them, but they are not suitable for my workplace

□ I know them, but I don’t have time to apply them

□ I know them and try to apply them or would like to apply them

2.11 Which handover tools do you know?

□ SBAR

□ ISOBAR

□ SAMPLE

□ MIST

**3. Handover Quality Questionnaire**

3.1 Are you satisfied with the current nursing handovers? (Mark only one):

□ Strongly disagree

□ Disagree

□ Somewhat agree

□ Strongly agree

3.2 Is the current handover method effective? (Mark only one):

(Effectiveness means the ability to fully achieve the desired outcome: optimal information exchange).

□ Strongly disagree

□ Disagree

□ Somewhat agree

□ Strongly agree

3.3 Is the current handover method efficient? (Mark only one):

(Efficiency means optimal information exchange within the allocated time).

□ Strongly disagree

□ Disagree

□ Somewhat agree

□ Strongly agree

3.4 Each nurse has their own way of giving handovers. (Mark only one):

□ Strongly disagree

□ Disagree

□ Somewhat agree

□ Strongly agree

(Mark only one response per row):

| The current handover method has resulted in... | Never | Sometimes | Often | Always |
| --- | --- | --- | --- | --- |
| 3.5 Medication errors |  |  |  |  |
| 3.6 Harm/injury to the patient |  |  |  |  |
| 3.7 Failure to deliver nursing care |  |  |  |  |
| 3.8 Delay in care delivery |  |  |  |  |
| 3.9 Miscommunication with other healthcare professionals |  |  |  |  |

3.10 Is the time allocated for handovers sufficient? (Mark only one):

□ Never

□ Rarely

□ Almost always

□ Always

3.11 Are there interruptions during handovers? (Mark only one):

□ Never

□ Rarely

□ Almost always

□ Always

| Express your opinion on each of the following aspects of handovers | Never | Sometimes | Often | Always |
| --- | --- | --- | --- | --- |
| 3.12 They ensure safe and adequate care |  |  |  |  |
| 3.13 They include the necessary information to set care priorities |  |  |  |  |
| 3.14 They provide a complete overview of the patient’s clinical situation |  |  |  |  |
| 3.15 The handover content ensures continuity of care |  |  |  |  |
| 3.16 The nurse receiving the handover is interested |  |  |  |  |
| 3.17 There is a discrepancy between verbal and written notes |  |  |  |  |
| 3.18 The nurse giving the handover includes irrelevant information |  |  |  |  |
| 3.19 The nurse giving the handover omits important information |  |  |  |  |

Supplementary Material 2.

| **Communality (*h_2_*)** |  |
| --- | --- |
| 3.1 Are you satisfied with the current nursing handovers? | 0.616 |
| 3.2 Is the current handover method effective? | 0.547 |
| 3.3 Is the current handover method efficient? | 0.654 |
| 3.4 Each nurse has their own way of giving handovers. | 0.286 |
| 3.5 Medication errors | 0.524 |
| 3.6 Harm/injury to the patient | 0.515 |
| 3.7 Failure to deliver nursing care | 0.540 |
| 3.8 Delay in care delivery | 0.477 |
| 3.9 Miscommunication with other healthcare professionals | 0.519 |
| 3.10 Is the time allocated for handovers sufficient? | 0.335 |
| 3.11 Are there interruptions during handovers? | 0.393 |
| 3.12 They ensure safe and adequate care | 0.609 |
| 3.13 They include the necessary information to set care priorities | 0.746 |
| 3.14 They provide a complete overview of the patient’s clinical situation | 0.620 |
| 3.15 The handover content ensures continuity of care | 0.705 |
| 3.16 The nurse receiving the handover is interested | 0.419 |
| 3.17 There is a discrepancy between verbal and written notes | 0.456 |
| 3.18 The nurse giving the handover includes irrelevant information | 0.468 |
| 3.19 The nurse giving the handover omits important information | 0.495 |
